# Biomechanical evaluation of different posterior fixation techniques for treating thoracolumbar burst fractures of osteoporosis old patients: a finite element analysis

**DOI:** 10.3389/fbioe.2023.1268557

**Published:** 2023-11-01

**Authors:** Guodong Zhang, Yukun Du, Guangzong Jiang, Weiqing Kong, Jianyi Li, Zhongjiao Zhu, Yongming Xi

**Affiliations:** ^1^ Department of Spinal Surgery, Tengzhou Central People’s Hospital, Tengzhou, China; ^2^ Department of Spinal Surgery, The Affiliated Hospital of Qingdao University, Qingdao, China

**Keywords:** thoracolumbar burst fractures, osteoporosis, biomechanical evaluation, posterior fixation, finite element analysis

## Abstract

**Objective:** To investigate the biomechanical characteristics of different posterior fixation techniques in treatment of osteoporotic thoracolumbar burst fractures by finite element analysis.

**Methods:** The Dicom format images of T10-L5 segments were obtained from CT scanning of a volunteer, and transferred to the Geomagic Studio software, which was used to build digital models. L1 osteoporotic burst fracture and different posterior fixation techniques were simulated by SolidWorks software. The data of ROM, the maximum displacement of fixed segment, ROM of fractured L1 vertebrae, the stress on the screws and rods as well as on fractured L1 vertebrae under different movement conditions were collected and analysed by finite element analysis.

**Results:** Among the four groups, the largest ROM of fixed segment, the maximum displacement of fixed segment and ROM of fractured vertebrae occurred in CBT, and the corresponding data was 1.3°, 2.57 mm and 1.37°, respectively. While the smallest ROM of fixed segment, the maximum displacement of fixed segment and ROM of fractured vertebrae was found in LSPS, and the corresponding data was 0.92°, 2.46 mm and 0.89°, respectively. The largest stress of screws was 390.97 Mpa, appeared in CBT, and the largest stress of rods was 84.68 MPa, appeared in LSPS. The stress concentrated at the junction area between the root screws and rods. The maximum stress on fractured vertebrae was 93.25 MPa, appeared in CBT and the minimum stress was 56.68 MPa, appeared in CAPS. And the stress of fractured vertebrae concentrated in the middle and posterior column of the fixed segment, especially in the posterior edge of the superior endplate.

**Conclusion:** In this study, long-segment posterior fixation (LSPF) provided with the greatest stability of fixed segment after fixation, while cortical bone screw fixation (CBT) provided with the smallest stability. Cement-augmented pedicle screw-rod fixation (CAPS) and combined using cortical bone screw and pedicle screw fixation (CBT-PS) provided with the moderate stability. CBT-PS exhibited superiority in resistance of rotational torsion for using multiple connecting rods. CAPS and CBT-PS maybe biomechanically superior options for the surgical treatment of burst TL fractures in osteoporotic patients.

## Introduction

Spinal fracture, accounting for about 5% of systemic fractures, is often caused by car accidents, high falls and other injuries (Cahueque et al., 2016). The thoracolumbar (TL) region is mostly involved due to the major contributing factor of the biomechanical stress transitional area from the semirigid thoracic spine to the mobile lumbar spine. Because of the high-energy trauma, nearly 10%–20% TL fractures are burst fractures. The compression and fracture of the vertebral body has the increasing injury risk of spinal cord and nerve to affect the patients’ life quality and labor ability (Hughes et al., 2021).

At present, conservative treatments including analgesia, bed rest and local immobilization are feasible for mild TL fracture patients without obvious neurological compromise and deformity. However, the delayed neurological deterioration of conservative treatments was reported up to 17% (Charles and Steib, 2015). Surgical interventions are most commonly recommended for burst TL fracture patients with neurological dysfunction and kyphosis. The main goal of surgical procedure is to provide patients with immediate spine stability, effectively neural decompression and kyphosis correction (Cook et al., 2021). With the aging of the population, the osteoporosis brings many challenges to treating the patient with burst TL fractures. The osteoporosis weakens the holding force of screws and obviously increases the risk of instrument failure. It was reported that the incidence of screw loosening was more than 60% of TL fracture patients with osteoporosis (Muratore et al., 2021).

There is still no consensus of the application guideline for different posterior fixation techniques in treatment of burst TL fractures with osteoporosis. Long-segment posterior fixation (LSPF) have advantages of better fixation strength and scattering stress, less possibility of spinal collapse and instrument failure. However, the extending fixation segment results in more surgical trauma and sacrifices more spinal motion segments, accelerating adjacent disc degeneration (Wang et al., 2019; Wu et al., 2019). The benefits of cement augmentation of pedicle screw fixation have been widely accepted. Compared with traditional screws, augmented by polymethylmethacrylate (PMMA) bone cement was able to improve the pullout resistance of screws in cancellous bone by 2–5 times. However, the complications including neural injury, pulmonary embolism and hypotension are still inevitable (Chevalier et al., 2021). Cortical bone screw is able to increase the screw holding force by adding more contact area between screw and cortical bone. Biomechanical experiments showed that CBT increase the axial stability of internal instrument nearly by 30% (Mai et al., 2016). Since firstly proposed by Santoni et al. (2009), the CBT technique is widely used in lumbar fusion with osteoporosis. However, it is rarely reported to be applied in burst TL fracture and the mechanical performance is still unclear. The cross trajectory technique by combined using cortical bone screw and pedicle screw showed the advantages of increasing fixation strength of pedicle screws, reducing the fixation segments as well as retaining the range of spine motion (Matsukawa et al., 2015), which may provide another reliable measurement to burst TL fracture.

To evaluate the biomechanical characteristics of different posterior fixation techniques, we simulated the finite element model of osteoporotic burst L1 fracture and conduct the finite element analysis of four different fixation techniques including cement-augmented pedicle screw fixation (CAPS), long-segmented pedicle screw fixation (LSPS), cortical bone screw fixation (CBT) and combined using cortical bone screw and pedicle screw fixation (CBT-PS). Under different movement conditions, data of ROM, maximum displacement of fixed segment, ROM of fractured L1 vertebrae, stress on instrument and fractured vertebrae were collected to compare the effectiveness of different posterior fixation techniques in treatment of burst TL fracture patients with osteoporosis, which provided theoretical basis for clinical application. The report is listed as follows.

## Materials and methods

### The establishment of intact models of T10-L5

One healthy 29-year-old male volunteer was enrolled in this study. The volunteer assigned the informed consent form before the study. This study was approved by the ethics committee of the affiliated hospital of Qingdao University (Ethics No. QYFYKYLL911411930).

The 64-slice spiral computed tomography scanner (Siemens, Germany) was used to scan this volunteer from the T10 to L5 vertebrae levels. The finite element (FE) model of the thoracolumbar from T10 to L5 was established as follows: 1) the CT images were scanned and the Dicom data was imported into Mimics Software 21.0 (Materialise, Leuven, Belgium) and the vertebral boundaries as regions of interest were identified with multiple images to form a 3-node triangular surface model. 2) The surface model was imported from SolidWorks (SOLIDWORKS Corporation, Boston, Massachusetts, United States) to further reconstruct a 3-dimensional solid model of the T10-L5 segment. 3) The solid model of the thoracolumbar spine was imported into Geomagic Wrap Software (Geomagic, Cary, North Carolina, United States) to optimize model shape, fit surface and build surface model. The nucleus pulposus and annular fibers were built separately. Intervertebral disc models of osteoporosis was simulated by the description of Sungwook Kang’s study (Kang et al., 2022). The volume ratio of the annulus fibrosus to the nucleus pulposus was set to 6:4. The thickness of vertebral cortical bone and endplate were set to 0.7 mm. The contact property of facet joint surface was set to tangential action without friction, and the initial gap of facet joint was set to 0.5 mm. The models of paraspinal ligaments (the anterior longitudinal ligament, the supraspinous ligament and the intertransverse ligament) were established by using Workbench (Ansys, Pittsburgh, Pennsylvania, United States). The parameters of material properties were used in this study shown on the Table 1. 4) The final FE models were imported to Abaqus FE analysis software (Abaqus, Simulia Corp., Providence, Rhode Island, United States) for analysis.

**TABLE 1:** Materials Property of the finite element model.

Element	Young’s modulus (MPa)	Poisson’s ratio	Cross-sectional area (mm^2^)
Cortical bone (osteoporosis)	8,040	0.3	—
Cancellous bone (osteoporosis)	34	0.2	—
Endplate	1,000	0.3	—
Cartilage	50	0.3	—
Annulus fibrosus	5	0.45	—
Nucleus pulposus	9	0.4	—
Screws and rods	110,000	0.28	—
Bone cement	3,000	0.4	—
Anterior ligaments	20	0.3	63.7
Posterior ligaments	20	0.3	20
Flavum ligaments	19.5	0.3	40
Intertransverse ligaments	12	0.3	40
Interspinal ligaments	15	0.3	30
Supraspinal ligaments	59	0.3	3.6
Capsular ligaments	75	0.3	60

### The establishment of thoracolumbar burst fracture models in osteoporotic condition

**FIGURE 1:**
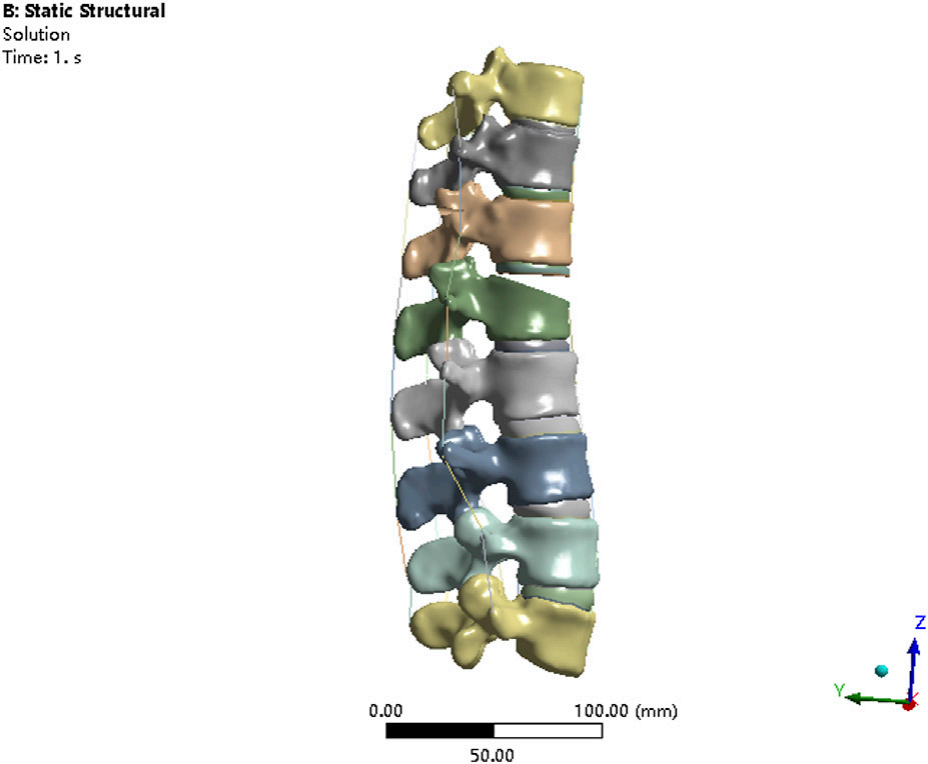
Finite element model of the spine with L1 burst fracture.

The V-shaped osteotomy of L1 vertebral body was performed by SolidWorks to simulate burst L1 fracture. The upper two-thirds of the anterior sponge bone of L1 vertebra was removed to weaken the vertebral strength, as described by Basaran et al. (2019). The anterior longitudinal ligament and posterior longitudinal ligament were discontinuous to fully simulate burst L1 vertebrae fracture (Figure 1). The material properties for the osteoporotic bony structures were reduced, compared with normal bony structures, by 66% of the elastic modulus for cancellous bone and by 33% for cortical bone, bony endplate, and posterior elements, as described by Guo et al. (2020).

### The finite element models of different internal fixations

The models of screws (55 mm × 45 mm/55 mm × 40 mm) and rods (diameter of 5 mm) were respectively constructed by SolidWorks software. Pedicle screws of thoracolumbar vertebrae were inserted by using the herringbone crest vertex technique. Based on the study brought by Santoni et al. (2009), the cortical bone screws were inserted.

**FIGURE 2:**
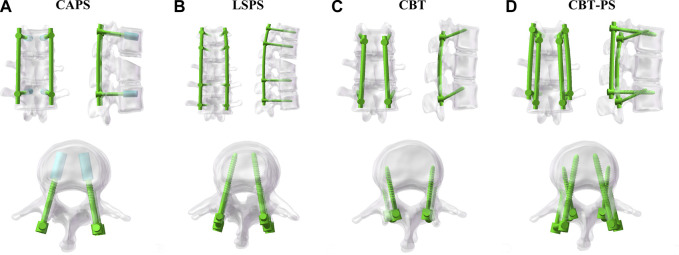
The four different posterior fixation models including CAPS, LSPS, CBT and CBT-PS.

Four finite element models of different posterior fixation techniques were simulated in this study. CAPS: Pedicle screws (55 mm × 45 mm) were inserted bilaterally in pedicles of T12, L2 vertebral arch. A cylindrical cement was built along each pedicle screw to the distal end and the volume of cement was set as 1 mL, as described by Wenhai Wang (Wang et al., 2014); LSPS: Pedicle screws (55 mm × 45 mm) were inserted bilaterally in pedicles of T11, T12, L2 and L3 vertebral arch; CBT:Cortical bone screws (55 mm × 40 mm) were inserted bilaterally in T12, L2 vertebra cortex; CBT-PS: Cortical bone screws (55 mm × 40 mm) and pedicle screws (55 mm × 45 mm) were inserted simultaneously in one vertebra cortex and vertebral arch in T12, L2 (Figure 2).

### Boundary conditions and loads

The lower edge of L5 vertebral body was fixed to limit the movement of L5 lower endplate in different directions. 500 N load was applied vertically above the T10 vertebral body, and 7.5 Nm was applied on the upper surface of the T10 vertebral body to simulate the movement including flexion, extension, bending and rotation. Range of motion (ROM), maximum displacement of fixed segment, ROM of fractured L1 vertebrae, stress distribution on constructs and fractured L1 vertebrae were analyzed under six movement conditions of flexion, extension, left bending, right bending, left rotation and right rotation, respectively.

## Results

### Validation of the intact T10-L5 finite element model

In order to evaluate the validity of T10-L5 intact model, 7.5 Nm torque was applied to the model to simulate the force under physiological load. Under the conditions of flexion, extension, left bending, right bending, left rotation and right rotation, the ROM of T10-L5 spinal intact model was 5.31°, 5.63°, 4.81°, 4.91°, 3.14°, 3.51°, respectively, which was comparable with the experimental data reported by Pflugmacher et al. (2004) and Basaran et al. (2019) to validate the rationality of the models (Table 2).

**TABLE 2:** Comparison between the normal spine model and models from previous studies.

ROM(°)			
	PresentStudy	Pflugmacher et al.	Basaran et al.
Flexion	5.31	5.3 ± 1.0	4.5 ± 0.9
Extention	5.63	5.7 ± 1.0	4.5 ± 0.9
Left bending	4.81	4.3 ± 0.6	4.2 ± 0.8
Right bending	4.91	4.3 ± 0.6	4.2 ± 0.8
Left rotation	2.14	2.1 ± 0.5	2.3 ± 0.6
Right rotation	2.51	2.1 ± 0.5	2.3 ± 0.6

Date from present study is comparable with the results from previous research.

### ROM of fixed segment in four FE fixation models

A significant decrease in ROM of fixed segment was found after fixation. The largest ROM in each group appeared under the movement of flexion and the smallest ROM was under extension.

**FIGURE 3:**
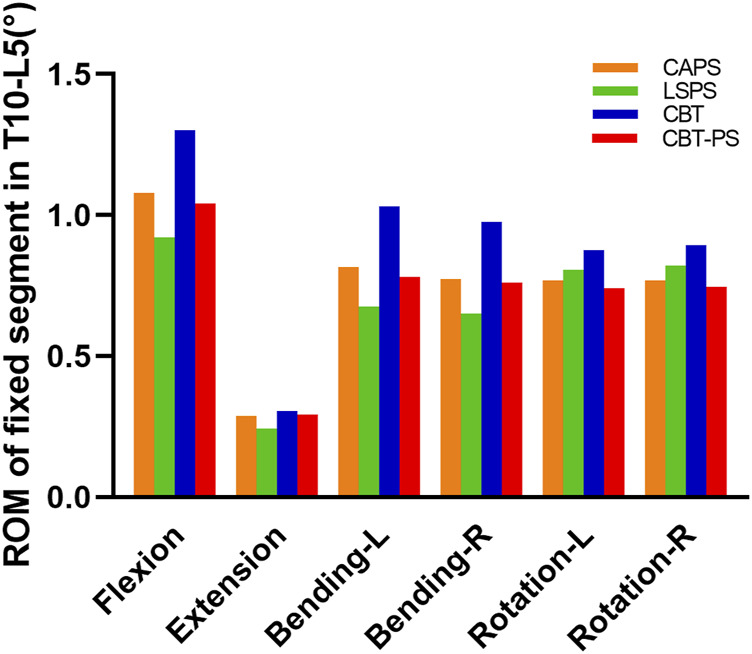
The ROM of fixed segment in T10-L5 of four FE models under different movement conditions including flexion, extension, bending and rotation.

The largest ROM of fixed segment was 1.3°, appeared in CBT. While the smallest ROM was 0.92°, appeared in LSPS. Among the four groups, CBT had the largest ROM in six directions and LSPS had the smallest ROM in directions of flexion, extension, left bending and right bending. Compared with CBT, CAPS had a decrease of 17.08% in flexion, 5.97% in extension, 20.78% in left bending, 20.58% in right bending, 12.28% in left rotation and 14.14% in right rotation. Compared with CBT, CBT-PS had a decrease of 20.11% in flexion, 4.19% in extension, 24.18% in left bending, 21.91% in right bending, 15.28% in left rotation and 16.43% in right rotation (Figure 3).

In addition, the decreased percentage of ROM after fixation in all groups was compared to assess the ability of stability restoration. CBT had the lowest decreased percentage in six directions (decreased by 67.01% in flexion, 92.61% in extension, 71.81% in left bending, 72.35% in right bending, 70.78% in left rotation, 66.74% in right rotation). The most obvious decreased percentage of ROM was found in LSPS in most directions except for axial rotation (decreased by 87.30% in flexion, 97.02% in extension, 90.45% in left bending, 90.97% in right bending).

The larger ROM and smaller decreased percentage after fixation in CBT means more abnormal movement compared with other techniques. So the stability of fixed segment in LSPS was best, while the worst stability occurred in CBT. The stability of CAPS and CBT-PS was between LSPS and CBT.

### The maximum displacement of fixed segments in four FE fixation models

The maximum displacement of fixed segment was defined as the max perpendicular distance between the posterior of upper and bottom vertebrae. In the research, we found that the maximum displacement of fixed segment decreased after fixation in all groups. The largest maximum displacement of fixed segment was 2.57 mm, appeared in CBT. The smallest maximum displacement was 2.46 mm, appeared in LSPS.

**FIGURE 4:**
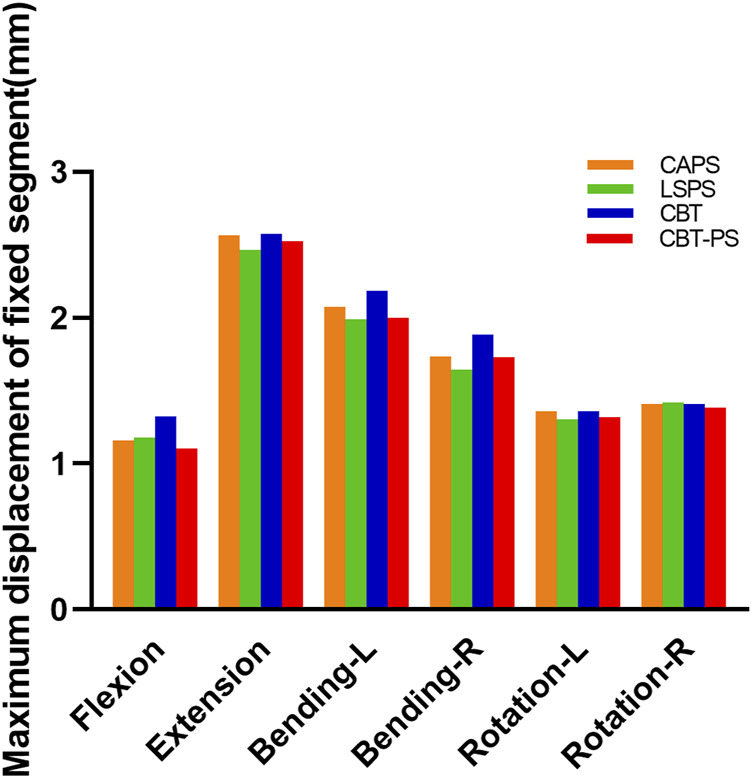
The maximum displacement of fixed segment of the four FE models.

LSPS has the smallest and CBT has the largest maximum displacement of fixed segment in most directions except for right rotation. Compared with CBT, CAPS had a decreased by 12.61% in flexion, 0.46% in extension, 4.9% in left bending, 7.9% in right bending, 0.9% in left rotation. Compared with CBT, CBT-PS had a decreased by 16.65% in flexion, 2.01% in extension, 8.27% in left bending, 8.28% in right bending, 3.05% in left rotation and 1.57% in right rotation. Interestingly, the largest maximum of fixed segment under right rotation occurred in LSPS (Figure 4).

The larger maximum displacement means the worse stability after fixation. In accordance with the results of ROM described above, the best stability occurred in LSPS and the worst was in CBT. The fixed segment in CAPS and CBT-PS had similar stability, which located between the stability of LSPS and CBT. However, it should be noted the largest maximum displacement in LSPS under the movement of right rotation may imply the disability to withstand excessive right rotation.

### ROM of fractured L1 vertebrae

ROM of fractured L1 vertebrae was recorded by measuring the Cobb angle variation of L1 vertebrae. The maximum ROM of fractured L1 vertebrae appeared under the movement of flexion, while the minimum ROM appeared under the movement of extension.

**FIGURE 5:**
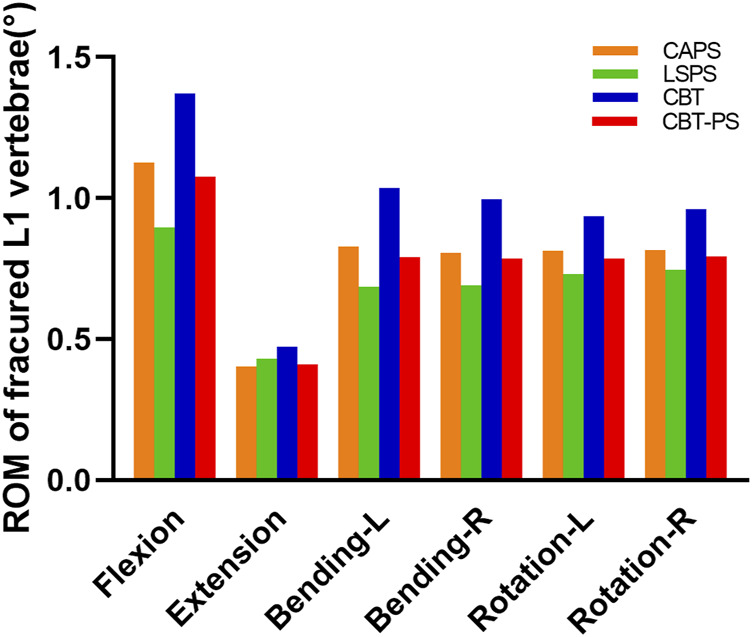
The ROM of fractured L1 vertebrae of the four FE models.

The largest ROM of fractured L1 vertebra was 1.37°, appeared in CBT. And the smallest ROM of fractured L1 vertebrae was 0.89°, appeared in LSPS. CBT had the largest ROM of fractured L1 vertebrae in six directions and LSPS had the lowest ROM in most directions except for extension. Compared with CBT, CAPS had a decrease by 17.2% in flexion, 14.78% in extension, 19.99% in left bending, 18.99% in right bending, 13.24% in left rotation and 15.02% in right rotation. Compared with CBT, CBT-PS had a decrease by 21.41% in flexion, 13.41% in extension, 23.66% in left bending, 20.89% in right bending, 16.20% in left rotation and 17.38% in right rotation (Figure 5).

### The von Mises stress on the screws and rods

The maximum von Mises stress of screws in CAPS, LSPS, CBT, CBT-PS was 191.45, 82.82, 390.97, and 214.66 MPa, respectively. The largest stress of screws was 390.97 MPa, appeared in CBT under the movement of flexion. Compared with CBT, the maximum von Mises stress of screws in LSPS, CAPS, CBT-PS decreased by 78.81%, 51.03% and 45.09%, respectively (Table 3).

**TABLE 3:** Maximum von Mises stress in the pedicle screws and rods.

		CAPS	LSPS	CBT	CBT-PS
Screws	Stress (MPa)	191.45	82.829	390.97	214.66
	Motion	Flexion	Left bending	Flexion	Flexion
	Level	T12	L3	T12	T12
Rods	Stress (MPa)	62.156	84.68	73.026	62.201
	Motion	Left bending	Right bending	Right bending	Left bending

**FIGURE 6:**
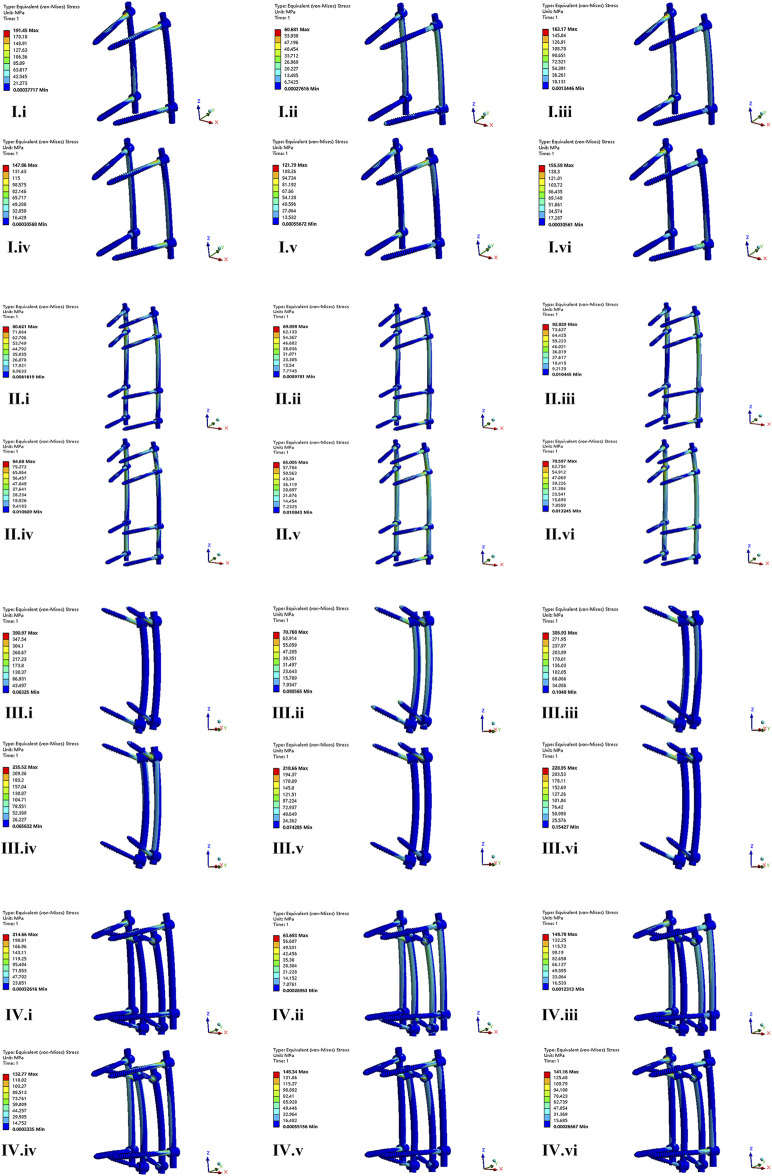
The nephogram of the maximum von Mises stress distribution of four FE models under different conditions. I–IV represent model of CAPS, LSPS, CBT, and CBT-PS; i–vi represent different direction of motion including flexion, extension, left bending, right bending, left rotation and right rotation.

For the rods, the maximum von Mises stress in CAPS, LSPS, CBT, CBT-PS was 62.15, 84.68, 73.02, and 62.20 MPa, respectively. The largest von Mises stress was 84.68 MPa, appeared in LSPS under the movement of right bending. Compared with LSPS, the maximum von Mises stress in CAPS, CBT-PS and CBT decreased by 26.59%, 26.54% and 13.76%, respectively (Figure 6).

The nephogram of the maximum von Mises stress in all models concentrated at the junction area between root of screws and the connecting rods.

### The von Mises stress on fractured L1 vertebrae body

The maximum von Mises stress of fractured L1 vertebral appeared under the movement of flexion and the minimum stress appeared under extension. The maximum von Mises stress in CAPS, LSPS, CBT, CBT-PS was 56.68, 82.55, 93.25, and 71.75 MPa, respectively. The largest maximum stress was 93.25 MPa, appeared in CBT under the movement of flexion. Compared with CBT, the maximum stress on fractured L1 vertebrae in CAPS, CBT-PS and LSPS decreased by 39.21%, 23.05% and 11.47%, respectively.

**FIGURE 7:**
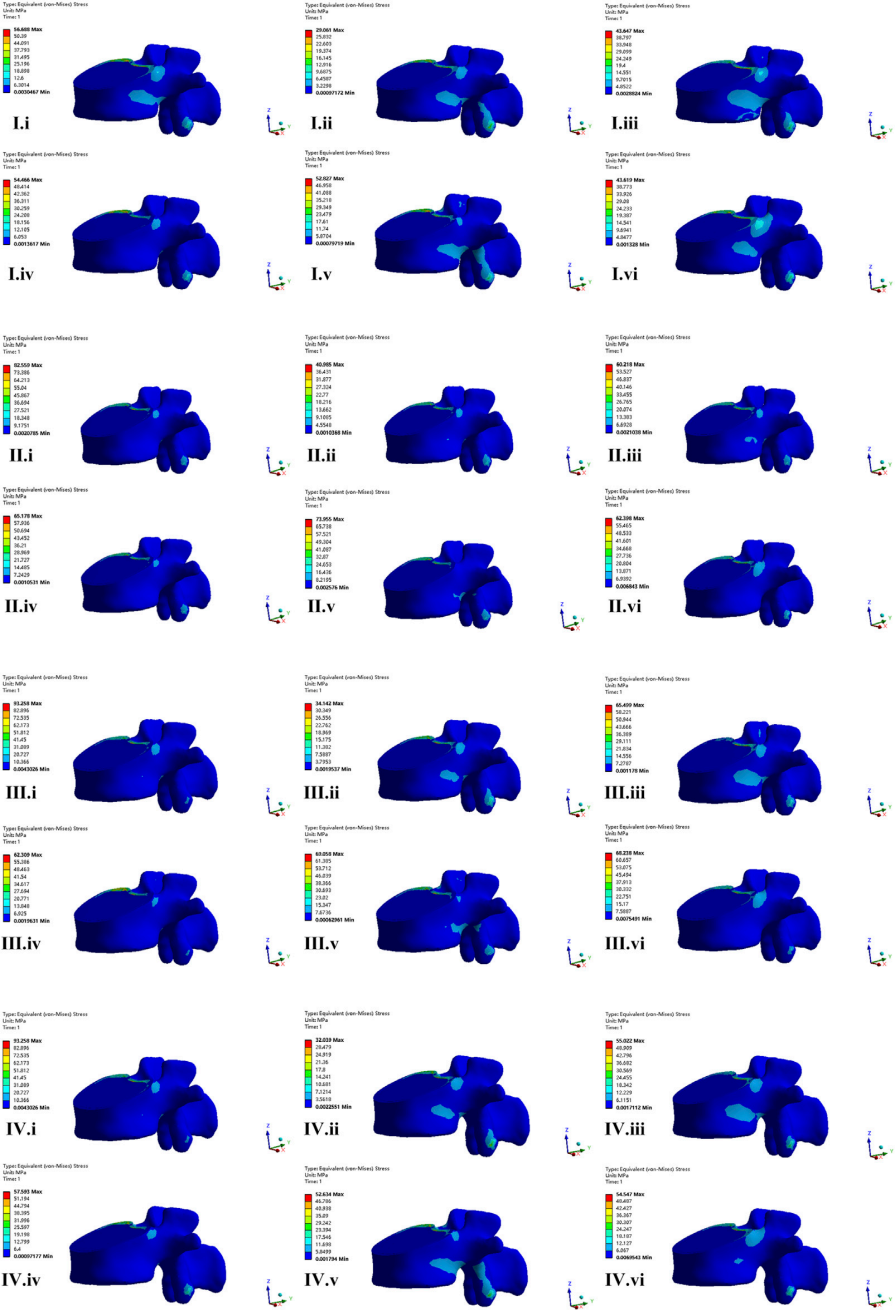
The nephogram of the von Mises stress distribution of fractured vertebrae body of four FE models. I–IV represent model of CAPS, LSPS, CBT, and CBT-PS; i–vi represent different direction of motion including flexion, extension, left bending, right bending, left rotation and right rotation.

The nephogram of the maximum von Mises stress of fractured L1 vertebrae was concentrated in the middle and posterior column of the fixed segment, especially in the posterior edge of the superior endplate (Figure 7).

## Discussion

As to the treatment of TL burst fractures, surgical intervention was indicated to provide patients with immediate stability, correction of kyphosis and effectively decompression of spinal cord and nerve. Posterior pedicle screw fixation system was regarded as an efficient method for the treatment of TL burst fractures. However, it should be cautious when the osteoporotic patients may suffer from instrument failure due to the negative influence of decreasing hold screw force (Liao, 2020). Maintaining adequate fixation strength is necessary to accelerate fracture healing and avoid pseudarthrosis. However, there is still no consensus of the application guideline for different posterior fixation techniques in treatment of burst TL fractures.

In present study, the FE analysis was conducted to compare biomechanical characteristics of different posterior fixation techniques to provide theoretical evidence for clinical application in treatment of burst TL fractures with osteoporosis. As described by Wang et al. (2014), we constructed cement-augmented fixation (CAPS) models. Briefly, the suitable PMMA cement (1 mL) was constructed along the pedicle screw to the distal end, and cement located in anterior and middle column of vertebrae mostly to avoid cement leakage. In his survey, it is observed that insertion of 1.0 cm^3^ cement causes pullout forces to increase by 50.7% and 60.8% for spherical and cylindrical cement volumes, respectively. When 2.5 cm^3^ of cement is inserted, the pullout forces are increased by approximately 116% and 120% for spherical and cylindrical cement zones, respectively (Wang et al., 2014). However, from our practical clinical experience, a bigger volume (2.5 mL) of cement is too larger to causing difficulty in cement insertion and inducing some complications, such as cement leakage and neurological deterioration. Based on the proof discussed above, we simulated cement augmentation models by inserting 1.0 cm^3^ cylindrical cement during the FE experiment. And we found that the fixation strength of pedicle screws increased after cement augmentation, which lead to a moderate stability of fixed segment. The balloon kyphoplasty (KP) with PMMA cement injection in fractured vertebrae is another method of cement augmentation. Shady Elmasry conducted an experiment to evaluate biomechanical perfomance of stand-alone KP, stand-alone percutaneous pedicle screws fixation (PPSF) and KP-augmented PPSF. However, the biomechanical superiority of KP-augmented PPSF over the stand-alone PPSF was not confirmed in that study (Elmasry et al., 2018).

Although CBT have been seen as one of the remedial surgical methods in osteoporotic patients, the mechanical result of CBT in our survey was not satisfactory. ROM and maximum displacement of fixed segment was largest under every movement condition, indicating the worst stability of fixed segment after fixation, which maybe caused by the insufficient support of anterior column. Matsukawa et al. found the stability after CBT fixation was worse under rotation and lateral bending movement condition (Sakaura et al., 2016). Although Sellin et al. (2018) demonstrated preferable results of the use of CBT to treat unstable traumatic thoracolumbar fractures, but this result based on fewer cases was more susceptible.

In addition, the result of our study showed the CBT-PS technique can supply with moderate stability of fixed segment and exhibit superiority in resistance of rotational torsion. The CBT-PS technique is a reliable and useful technique to reinforce the fixation strength for creating multiple points of fixation within a vertebrae. Combined using pedicle screw and cortical bone screw simultaneously can make up the shortcomings of insufficient fixation strength of CBT (Ueno et al., 2013). Michal Szczodry in his survey pointed out that the increased cortical purchase (ICP) insertion technique results in same biomechanical performance and same accuracy as the straightforward technique by using guidance (Szczodry et al., 2018). However, the difficulty of inserting CBT and pedicle screw is a common problem confronted in practical use. At first, the pedicle size need to be large enough to accommodate two screws in one pedicle. In our study, the selected pedicle screws were 5.5 mm in diameter and 50 mm in length; the CBT screws were 5.5 mm in diameter and 40 mm in length, which was in accordance with Keitaro Matsukawa’s survey (Matsukawa et al., 2015). And then, choosing the ideal screw path for the optimal fixation plays an important role in the cross-trajectory technique. With the development of technology, difficulties in inserting screw safely and pricisely can be overcomed with the use of appropriate technologies. In a cadaver study, individualized surgical guide templates for double-trajectory screw placement were designed and the result was satisfactory (Zhao et al., 2021).

Previous studies have shown that the pedicle screw fixation system converts 50% axial pressure of the vertebral body into the pressure on the screw and connecting rod in lumbar fusion (Spiegl et al., 2022). In burst TL fractures, the more pressure on the screw caused by the loss of anterior and middle column support increased the risk of instrument failure. In this study, the largest stress on screws occurred in CBT, and the smallest stress was in LSPS. The worse stability in CBT means more abnormal movement, which exert more stress on instruments. Adding additional screws and augmentation with cement can scatter the screw stress effectively. For the rods, the largest stress occurred in LSPS. Extending fixed segment lead to the increase of stiffness and local stress concentration. What’s more, we found that the maximum stress of the screw concentrate at junction area between the root of the screw and the connecting rod, which is consistent with previous study (Wu et al., 2019). Although the peak stress of screw and connecting rod do not exceed the 529 MPa endurance limit of titanium alloy (Wycisk et al., 2014), the spine is a relatively active unit and local fretting exert the continuous stress of internal instrument increasing the risk of fatigue fracture.

The axial compression pressure was delivered downward to the fractured vertebral body. The excessive stress on the fractured vertebral body has the negative effect on fracture healing. In this research, the stress on fractured vertebrae under flexion movement was bigger than other movement condition. The largest stress on fractured vertebrae occurred was found in CBT, caused by the poor ability to restore stability of fixed segment. CAPS has the smallest stress on fractured vertebrae, which is resort to the fact more points of fixation on vertebral make the stress more dispersed rather than concentrated. In addition, bone cement has inherent advantages of bone induction for bone growth, which is beneficial to recovery from burst fracture (Cai et al., 2023).

FE analysis is a reliable and helpful method to predict mechanical strength and dynamic characteristics of simulated constructs. However, there are many aspects of influencing factors in the process of FE analysis. Osteoporosis is clinically related to an age-related degeneration process, cell density, nutrition level, proteoglycans (PGs), water contents, as well as volume change in the disc decreased over the progression of degeneration across decades, as discussed by Mallory Volz (Volz et al., 2021). In our study, we built osteoporotic models of intervertebral disc, and the model was validated with others’ literature. In addition, instrument properties such as metal material, craft of surface coating may have an effect on the result of FE analysis. Patrick A. Massey compared the biomechanical performance of nitinol memory metal rods and titanium rods. He found that nitinol trended toward superior fatigue resistance, but there was no significant difference in nitinol versus titanium construct fatigue resistance (Massey et al., 2021). In an experiment of SWEETU PATEL, the nanotubes surface were inserted at Ti6Al4V rod, and the stability between the bone-implant interface was promoted (Patel et al., 2015).

There are some limitations in our study. First, the finite element model is constructed by obtaining the CT data of a single patient and hardly represent the biomechanics of different ages and genders. Second, the current finite element model does not include the spinal muscle system, which plays an important role in maintaining the stability of the spine. At last, increasing the diameter and length of the screw can potentially produce larger pullout forces but may also increase the risk of fracturing the surrounding fragile bone (Solitro et al., 2019). The mechanical performance was not included in this study. The further biomechanical experiments in cadaver studies and clinical cohort studies would be needed to be performed to prove the results of this study.

## Conclusion

In this study, long-segment posterior fixation (LSPF) provided with the greatest stability of fixed segment after fixation, while cortical bone screw fixation (CBT) provided with the smallest stability. Cement-augmented pedicle screw-rod fixation (CAPS) and combined using cortical bone screw and pedicle screw fixation (CBT-PS) provided with the moderate stability. CBT-PS exhibited superiority in resistance of rotational torsion for using multiple connecting rods. CAPS and CBT-PS maybe biomechanically superior options for the surgical treatment of burst TL fractures in osteoporotic patients.

## Data Availability

The raw data supporting the conclusion of this article will be made available by the authors, without undue reservation.
